# Antibiotics and resistance: the two-sided coin of the mycobacterial cell wall

**DOI:** 10.1016/j.tcsw.2020.100044

**Published:** 2020-09-02

**Authors:** Sarah M. Batt, Christopher E. Burke, Alice R. Moorey, Gurdyal S. Besra

**Affiliations:** Institute of Microbiology and Infection, School of Biosciences, University of Birmingham, Edgbaston, Birmingham B15 2TT, UK

**Keywords:** *Mycobacterium tuberculosis*, Cell wall, Arabinogalactan, Mycolic acids, Lipoarabinomannan

## Abstract

*Mycobacterium tuberculosis*, the bacterium responsible for tuberculosis, is the global leading cause of mortality from an infectious agent. Part of this success relies on the unique cell wall, which consists of a thick waxy coat with tightly packed layers of complexed sugars, lipids and peptides. This coat provides a protective hydrophobic barrier to antibiotics and the host’s defences, while enabling the bacterium to spread efficiently through sputum to infect and survive within the macrophages of new hosts. However, part of this success comes at a cost, with many of the current first- and second-line drugs targeting the enzymes involved in cell wall biosynthesis. The flip side of this coin is that resistance to these drugs develops either in the target enzymes or the activation pathways of the drugs, paving the way for new resistant clinical strains. This review provides a synopsis of the structure and synthesis of the cell wall and the major current drugs and targets, along with any mechanisms of resistance.

## 1 Introduction

**Fig. 1:**
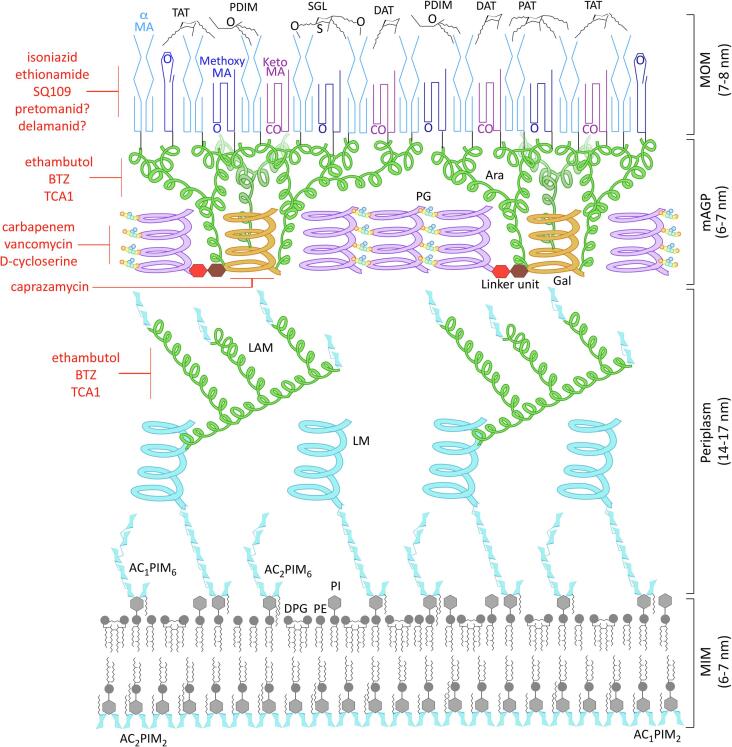
The cell wall of *Mycobacterium tuberculosis*. The inner leaflet of the plasma membrane contains a high quantity of Ac_1_/Ac_2_PIM_2_ (tri- and tetra-acylated phosphatidyl-*myo*-inositol-dimannoside), while the outer membrane has Ac_1_/Ac_2_PIM_6_ (tri- and tetra-acylated phosphatidyl-*myo*-inositol-hexamannoside), along with the more usual phospholipids, DPG (diphosphatidylglycerol), PE (phosphatidylethanolamine) and PI (phosphatidylinositol); the methyl groups of the unique tuberculostearic acids of mycobacteria are depicted here (Minnikin et al., 2015). Also anchored into the plasma membrane are LM (lipomannan) and LAM (lipoarabinomannan), which project out into the periplasm; the mannose sugars and mannan domains are coloured light blue and the branched arabinan is green. According to the ‘scaffold model’, the glycan back bone (purple) of the PG (peptidoglycan) forms a matrix of helices orientated perpendicular to the plasma membrane (Dmitriev et al., 2000). These surround the AG (arabinogalactan) and LAM (lipoarabinomannan) and are connected by the peptide cross-links (coloured circles: orange = L-alanine, yellow = D-*iso*glutamine, green = *meso*-diaminopimelate and blue = D-alanine). The PG is connected to the base of the Gal (galactan; orange) *via* a unique rhamnose-N-acetylglucosamine linker. Three highly branched Ara (arabinan; green) domains project from the base of the Gal towards the MA layer (mycolic acids; dark blues and purples), which is covalently attached to most of the non-reducing ends of the Ara and forms the inner layer of the MOM (mycobacterial outer membrane). The PG, AG and MA make up the mycolylarabinogalactan-peptidoglycan complex (mAGP). The free lipids of the outer leaflet consist of PDIM (phthiocerol dimycocerosates); DAT, TAT, PAT and SGL (di-, tri- and penta-acyl trehalose and sulfated trehalose glycolipids) (Jankute et al., 2015, Minnikin et al., 2015). The diagram is roughly to scale using dimensions obtained from cryo-electron microscopy (Zuber et al., 2008). The main current and pipe-line drugs targeting the biosynthesis/transport pathways of the cell wall are shown in red. (For interpretation of the references to colour in this figure legend, the reader is referred to the web version of this article.)

*Mycobacterium tuberculosis* (*Mtb*), the causative agent of tuberculosis, has a mortality rate of over 1.5 million a year (World Health Organisation, 2019). A crucial part of this pathogenicity is the extremely unusual cell wall of mycobacteria, and as such many of the current antibiotic regimes target essential enzymes involved in its synthesis. The first-line drug regimen is a combination of antibiotics, consisting of ethambutol, isoniazid, rifampicin and pyrazinamide (World Health Organization, 2017). Since these drugs have been used for more than 60 years, multi-drug resistant strains (MDR) have developed, with mutations in the target enzymes or drug activation pathways. Second-line drugs, which include capreomycin, ethionamide and streptomycin, are the next level of treatment for those with resistant strains (World Health Organisation, 2011). However, extensively drug resistant strains (XDR), which are additionally resistant to second-line drugs, are also emerging (World Health Organisation, 2011). This has resulted in a surge of research into mycobacteria, in the hopes of finding new effective drugs and targets. As many of the current drugs, and those under development, target the cell wall (see Fig. 1 for examples in red), an understanding of the complex biosynthesis pathways and mechanisms of drug inhibition and resistance, is a valuable part of this research.

The cell envelope of mycobacteria (Fig. 1) is a waxy, hydrophobic coat, with a high percentage of lipids (40%) (Asselineau and Lederer, 1950), imparting this pathogen with an effective barrier to antibiotics (Brennan and Nikaido, 1995, Minnikin, 1982) and the host’s immune system (Harding and Boom, 2010, Murry et al., 2009, Reed et al., 2004), as well constituting key virulence factors (Fratti et al., 2003, Glickman et al., 2000, Maeda et al., 2003, Quigley et al., 2017, Schlesinger et al., 1994). Though classified as a Gram-positive, mycobacteria actually possess an outer membrane or “MOM” (mycobacterial outer membrane), which is not typical of Gram-negatives either. This MOM consists of an inner layer of mycolic acids (MAs) and an outer leaflet of free-lipids (Fig. 1) (Barksdale and Kim, 1977, Christensen et al., 1999, Minnikin, 1982, Zuber et al., 2008). The MA layer is covalently linked to the arabinogalactan (AG), a domain of heavily branched arabinose chains connected to a galactan trunk, which in turn is attached to the peptidoglycan, forming the mycolyl-arabinogalactan-peptidoglycan (mAGP) complex (Barry et al., 2007, Brennan and Nikaido, 1995, Crick et al., 2010, Crick et al., 2001). Also present are the lipoglycans, lipomannan (LM) and lipoarabinomannan (LAM), which are anchored within the inner membrane and project out into the periplasmic space, though there is some evidence to suggest that they also reside in the lipids of the MOM (Minnikin et al., 2015, Ortalo-Magne et al., 1996, Pitarque et al., 2008, Sani et al., 2010). The inner membrane of mycobacteria is also rather unconventional, containing a high proportion of phosphatidyl-*myo*-innositol mannosides (PIMs), which serve to improve the membrane’s stability and barrier to drugs (Bansal-Mutalik and Nikaido, 2014).

This review will discuss the structures and biosynthesis of the major components of the *Mtb* cell envelope, including the current drugs that target their synthesis and transport, and in turn the resistance mechanisms that have developed. Interesting drugs in the pipe-line and new target enzymes will also be considered, along with the changing aspects of research into drug discovery.

## 2 The cell wall core

### 2.1 Peptidoglycan structure

**Fig. 2:**
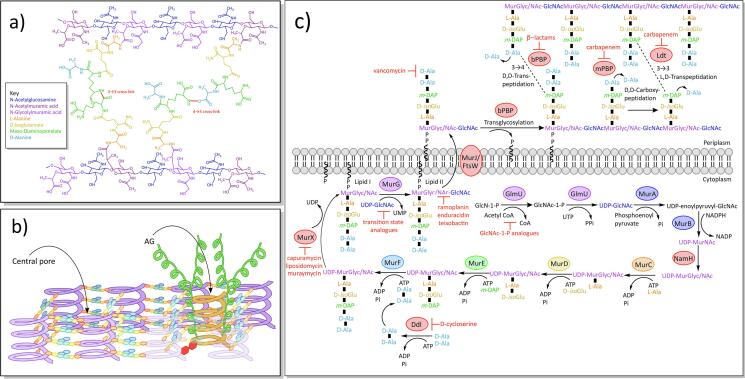
Structural features of peptidoglycan and biosynthesis A) Chemical structure of peptidoglycan (see key for details). B) Diagram of the ‘scaffold model’ of peptidoglycan. The glycan (purple) of the peptidoglycan forms a matrix of helices orientated perpendicular to the plasma membrane, joined together by peptide cross-links and forming central pores to fit other structures, such as arabinogalactan (AG; orange and green helices) (Dmitriev et al., 2000). C) Biosynthesis of peptidoglycan. (For interpretation of the references to colour in this figure legend, the reader is referred to the web version of this article.)

**Fig. 3:**
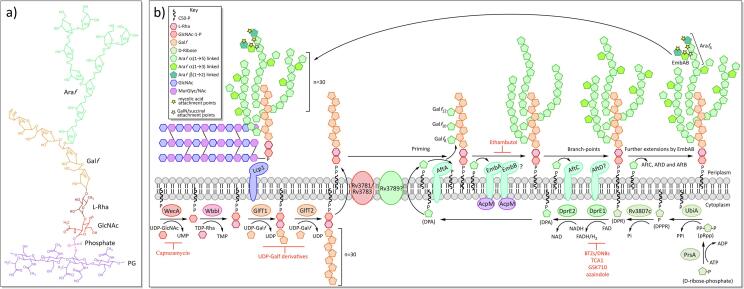
Structural features of arabinogalactan and biosynthesis A) Chemical structure of arabinogalactan. B) Biosynthesis of arabinogalactan and the rhamnose-N-acetylglucosamine linker unit.

Peptidoglycan (PG) is common to all bacteria and provides crucial mechanical strength to the cell wall in order to resist internal cellular hydrostatic pressure and maintain shape, any disruption of which leads to bactericidal cell lysis (Vollmer et al., 2008). While for most bacteria, the peptidoglycan also provides a major anchoring point on the exterior of the cell for further modifications, uniquely in mycobacteria, it forms the base for the entire mycobacterial outer membrane complex, the mAGP (Brennan and Nikaido, 1995). Structurally, PG consists of long β(1 → 4)-linked glycan backbone chains of repeating N-acetylglucosamine (GlcNAc) and N-acetylmuramic acid residues (MurNAc), cross-linked together *via* peptide side-chains of five amino acids, to form a mesh-like macromolecule (Fig. 2A) (Brennan and Nikaido, 1995). Though a common A1γ subtype found in many bacteria including *Escherichia coli (E. coli)* (Brennan and Nikaido, 1995, Schleifer and Kandler, 1972), mycobacterial PG has several modifications to the structural features. The muramic acid residues, for instance, are present as a mixture of both the typical MurNAc and a hydroxylated derivative, N-glycolylmuramic acid (MurGlyc), in mycobacteria (Mahapatra et al., 2005). The reason for this has yet to be determined, however it is thought that the glycolyl derivative has the potential to form additional hydrogen bonds, thereby increasing the strength of the PG (Brennan and Nikaido, 1995). The sequence of the mycobacterial peptide linker is L-alanine (L-Ala), D-*iso*glutamate (D-*iso*Glu), *meso*-diaminopimelate (*m-DAP*), D-alanine (D-Ala) and D-Ala (Brennan and Nikaido, 1995); further modifications of these amino acids include the amidation of the free carboxylic acids of D-*iso*Glu, *m*-DAP and the terminal D-Ala, though the significance of this is not known (Mahapatra et al., 2005). Another deviance from the usual PG structure is the cross-linking of the peptide chains, which consist mostly of 3 → 3 linkages between two central *m-DAP* residues in mycobacteria, with fewer of the 3 → 4 *m-DAP* to D-Ala linkages common to most prokaryotes (Kumar et al., 2012, Lavollay et al., 2008). Mycobacteria also have significantly more peptide cross-links (up to 70–80%) compared to other species, such as *E. coli* (50%), which provides additional mechanical strength to the mesh-like structure (Matsuhashi, 1966, Vollmer and Holtje, 2004). The PG is attached to the arabinogalactan (AG) *via* a unique linker unit in mycobacteria, α-L-rhamnopyranose-(1 → 3)-α-D-GlcNAc(1 → P), which forms the connection through a phosphodiester bond to the 6-OH of the muramic residues in the glycan backbone (Fig. 3A) (McNeil et al., 1990).

Earlier models of the 3D structure of PG within the cell envelope depicts the layers of the glycan backbone lying along the same plane as the plasma membrane (Ghuysen, 1968), orientated perpendicularly to the MAs. However, this model could theoretically result in gaps within the structure of the PG, and a recent ‘scaffold model’ instead positions the glycan back-bones as helices, perpendicular to the plasma membrane (Fig. 2B) (Dmitriev et al., 2000). These helices are proposed to form a matrix with central spaces or ‘pores’ within each set of four peptide-linked helices, that could surround an AG moiety or other structure such as LAM (Dmitriev et al., 2000). This idea sustains biochemical (Besra and Brennan, 1997), chemical (Besra et al., 1995) and EM data (Rastogi et al., 1991) and has since been further supported by Nuclear Magnetic Resonance data (Meroueh et al., 2006).

### 2.2 Peptidoglycan biosynthesis

PG biosynthesis (Fig. 2C) begins in the cytoplasm with GlmU (Rv1018c), an enzyme with two sequential functions in the synthesis of uridine diphosphate-N-acetylglucosamine (UDP-GlcNAc) (Zhang et al., 2009). As an acetyltransferase, GlmU first transfers an acetyl group from acetyl-coenzyme A (acetyl-CoA) to glucosamine-1-phosphate, producing N-acetylglucosamine-1-phosphate (Zhang et al., 2009), which is further modified by GlmU’s uridyltransferase activity and UTP, into UDP-GlcNAc (Zhang et al., 2009). This is either incorporated into Lipid II as mentioned later, or converted into UDP-MurNAc, a process that is carried out by MurA (Rv1315) and MurB (Rv0482). MurA, a UDP-GlcNAc enolpyruvyl transferase, first transfers enolpyruvate, from phosphoenolpyruvate, to the UDP-GlcNAc, forming UDP-N-acetylenolpyruvylglucosamine (De Smet et al., 1999). MurB (Rv0482) is a UDP-N-acetylenolpyruvylglucosamine reductase that next reduces the enolpyruvate group, using the cofactor NADPH, to UDP-MurNAc (Eniyan et al., 2018). In mycobacteria, much of the UDP-MurNAc is hydroxylated to UDP-MurGlyc by NamH, a UDP-MurNAc hydroxylase (Mahapatra et al., 2005, Raymond et al., 2005). The penta-peptide side chain is added to the UDP-MurGlyc/NAc by the ATP-dependant Mur ligases: MurC, MurD and MurE (Rv2152c, Rv2155c and Rv2158c), which ligate L-Ala, D-*iso*Glu and *m-*DAP sequentially (Kurosu et al., 2007, Munshi et al., 2013). The peptide sidechain is completed by MurF (Rv2157c) adding a D-Ala:D-Ala dipeptide subunit (Munshi et al., 2013), which is synthesised by Ddl, a D-Ala-D-Ala ligase (Rv2981c) (Bruning et al., 2011). This muramyl-pentapeptide unit, known as the Park’s nucleotide (Chen et al., 2016), is then anchored into the inner membrane by MurX (Rv2156c) (a phospho-N-acetylmuramoyl-pentapeptide transferase, also known as MraY), which conjugates a decaprenyl phosphate (C_50_-P), producing Lipid I (Chen et al., 2016, Kurosu et al., 2007). The glycosyltransferase, MurG (Rv2153c), then converts Lipid I to Lipid II, transferring a UDP-GlcNAc to the muramic acid residue of Lipid I, with β(1 → 4) linkage (Lecreulx et al., 1991).

Lipid II is next translocated across the inner membrane to the periplasm. The protein responsible for this was initially thought to be either MurJ (Rv3910) or the Shape, Elongation, Division, Sporulation (SEDS) protein, FtsW (Rv2154c) (Leclercq et al., 2017, Mohammadi et al., 2011, Ruiz, 2015, Ruiz, 2008, Sham et al., 2014). Recent evidence points to MurJ as the more likely flippase (Zheng et al., 2018) and to FtsW as a PG glycosyl transferase (Emami et al., 2017, Meeske et al., 2016, Taguchi et al., 2019). Native mass spectroscopy has demonstrated *in vitro* binding of the *E. coli* MurJ to Lipid II (Bolla et al., 2018) and the crystal structure of MurJ from *Thermosipho africanus* (MurJ_TA_) has also provided evidence of MurJ’s role in Lipid II transport (PDB IDs: 5T77, 6NC6, 6NC7, 6NC8 and 6NC9) (Kuk et al., 2019, Kuk et al., 2017). MurJ_TA_ was shown to have a cytoplasmic entrance and a periplasm exit, as well as a cytoplasmic gate and a central cavity that crystallised in both inward- and outward-open conformations, demonstrating how MurJ might flip Lipid II (Kuk et al., 2019, Kuk et al., 2017). Electron density, hypothesised to be Lipid II, was present in the portal and central cavity of the inward configuration of MurJ_TA_; this portal is thought to regulate the entry of Lipid II into the central cavity of MurJ (Kuk et al., 2019).

Once on the exterior face of the membrane, Lipid II is polymerised into a chain of PG by the mono-functional and bi-functional penicillin binding proteins (PBPs) (Sauvage et al., 2008). The bi-functional PBPs, PonA1 (Rv0050), PonA2 (Rv3682) and PonA3, have both a transglycosylase domain for polymerising the glycan backbone, and a D,D-transpeptidase domain to form the 3 → 4 crosslinks between *m*-DAP and D-Ala of adjacent peptide side-chains, cleaving the terminal D-Ala from one side-chain (Kieser et al., 2015, Patru and Pavelka, 2010). In mycobacteria, however, 80% of the crosslinks are the non-classical 3 → 3 linkage (Kumar et al., 2012, Lavollay et al., 2008). These cross-links are formed by the combined activities of the D,D-carboxypeptidases of the monofunctional PBPs, which remove the terminal D-Ala from one sidechain (Pandey et al., 2018), followed by the activity of the L,D-transpeptidases (Ldts), which cleaves the next D-Ala and forms a 3 → 3 crosslink between two *m*-DAP residues of nearby sidechains (Laponogov et al., 2009). There are two Ldts encoded in the *Mtb* genome, termed Ldt_Mt1_ (LdtA; Rv0116c) and Ldt_Mt2_ (LdtB; Rv2518c) (Gupta et al., 2010), which are structurally unrelated to the PBPs and contain an active-site cysteine instead of serine (Biarrotte-Sorin et al., 2006, Mainardi et al., 2005).

### 2.3 Drugs targeting peptidoglycan synthesis and resistance mechanisms

Due to the structural importance of PG, it is an attractive target for antimicrobials. Indeed, part of the mammalian innate immune system is the production of lysozyme, an enzyme that hydrolyses the β(1 → 4) links between GlcNAc and MurGlyc/NAc in the glycan backbone (Berger and Weiser, 1957, Chipman and Sharon, 1969); mycobacteria have a measure of resistance to lysozyme, which is conferred by the modification of MurNAc to MurGlyc (Raymond et al., 2005). The most well-known antibiotics that target the biosynthesis of PG are the β-lactams, analogues of D-Ala-D-Ala that bind irreversibly to the active site of the PBP’s transpeptidase domain (Kurz and Bonomo, 2012), preventing 3 → 4 peptide crosslinking. Whilst β-lactams are highly active against both Gram-positive and Gram-negative bacteria, in mycobacteria, most of the peptide crosslinking is 3 → 3 and constructed by the structurally distinct Ldts, which are not inhibited by most β-lactams (Kurz and Bonomo, 2012, Mainardi et al., 2005). Additionally, in many bacteria, resistance to β-lactams has developed in the form of β-lactamases, which destroy the lactam ring. Mycobacteria are no exception to this and express the enzyme, BlaC (Rv2068c), a broad spectrum and highly active β-lactamase (Wang et al., 2006). These factors have meant that β-lactams were not considered as a treatment for mycobacteria for a long time. Recently though, a combinatorial regime has been developed for *Mtb* that utilises carbapenem, a β-lactam able to inhibit the Ldts and D,D-carboxypeptidases of mycobacteria (Cordillot et al., 2013, Dubée et al., 2012, Hugonnet et al., 2009, Kumar et al., 2012), with a β-lactamase inhibitor, clavulanic acid, which inhibits BlaC (Hugonnet et al., 2009, Hugonnet and Blanchard, 2007, Tremblay et al., 2008). This regime is highly effective against drug-resistant strains of *Mtb* (Kurz and Bonomo, 2012, Ramón-Garciá et al., 2016). Unfortunately, research has suggested that there are point mutations in BlaC that could enable this β-lactamase to hydrolyse the clavulanic acid (Soroka et al., 2015).

D-cycloserine is a second-line drug, active against drug resistant mycobacteria (MDR and XDR) (World Health Organisation, 2018), that targets the biosynthesis of PG. As an analogue of D-Ala, it is a competitive inhibitor for both alanine racemase (Alr), the enzyme that converts alanine between the L- and D- forms (Halouska et al., 2007), and Ddl, which forms the terminal D-Ala:D-Ala dipeptide of the PG’s peptide sidechains (Bruning et al., 2011, Halouska et al., 2014). While both enzymes are inhibited by D-cycloserine, Alr inhibition is not lethal (Halouska et al., 2007) and the bacteriostatic effect in mycobacteria is caused by the inhibition of Ddl (Halouska et al., 2014, Prosser and de Carvalho, 2013); though Alr inhibition may indirectly enhance D-cycloserine potency by decreasing the available D-Ala to compete for Ddl (Halouska et al., 2014). D-cycloserine is an attractive drug, with extremely low levels of spontaneous resistance in clinical strains, which could be explained by the observed *in vivo* fitness costs of the few resistance mutations that have arisen in *Mtb* (Evangelopoulos et al., 2019). Most known resistance mechanisms occur in Alr, including a point mutation (aspartic acid-322 to asparagine) (Coll et al., 2018), and a promotor mutation that leads to Alr overexpression; this latter mutant has increased levels of D-Ala, which outcompete the D-cycloserine for Ddl binding (Halouska et al., 2014, Halouska et al., 2007, Nakatani et al., 2017).

Vancomyin, a glycopeptide that binds to the terminal D-Ala:D-Ala and prevents cross-linking of the pentapeptide side chains (Reynolds, 1989), is mainly used to treat Gram-positive bacteria due to its inability to cross the outer membrane of Gram-negatives and mycobacteria. However, vancomycin could prove useful in mycobacteria if used in combination with inhibitors of cell wall synthesis enzymes, such as ethambutol (Arain et al., 1994, Soetaert et al., 2015). Ramoplanin (Wu et al., 2015), enduracidin and teixobactin (Ling et al., 2015, Piddock, 2015) are inhibitors that bind directly to Lipid II, preventing transglycosylation of the glycan backbone. Ramoplanin and enduracidin are structurally related, though an additional mannose moiety on ramoplanin enhances solubility (Wu et al., 2015). Teixobactin is a peptide-like antibiotic, derived from uncultured soil bacteria, that is active against Gram-positive bacteria and *Mtb,* with low levels of spontaneous resistance (Ling et al., 2015, Piddock, 2015).

The bi-functional enzyme, GlmU, which is involved in UDP-GlcNAc synthesis, is essential (Zhang et al., 2008), and several inhibitors have been examined, including substrate analogues (Li et al., 2011) and the natural compounds dicumarol and coumarin (Han et al., 2019). There are a number of naturally occurring nucleoside antibiotics, including capuramycin, caprazamycin, liposidomycin and muraymycin, that target MurX, the essential translocase that produces Lipid I (Boyle and Donachie, 1998, Dini, 2005). Several derivatives of capuramycin have been made, some of which are active against non-replicating *Mtb* (Reddy et al., 2008, Siricilla et al., 2015). The most successful derivative is SQ641, which has been through pre-clinical trials, though its poor aqueous solubility is disadvantageous (Pstragowski et al., 2017, Reddy et al., 2008).

### 2.4 Arabinogalactan structure

Arabinogalactan (AG) is a tree-like structure composed of a galactose trunk with arabinose branches, covalently attached to the PG *via* a rhamnose-GlcNAc linker unit at the base of the trunk (Fig. 3A) (Lechievalier and Lechievalier, 1970, McNeil et al., 1987). The main trunk structure consists of approximately 30 β-D-galactofuranose residues (Gal*f*) with alternating β(1 → 5) and β(1 → 6) linkages (Daffé et al., 1990). This is elaborated with three arabinofuranose (Ara*f*) branches, all of which are attached to the 6-linked Gal*f* residues at the 8th, 10th, and 12th positions of the trunk (Alderwick et al., 2005, Besra et al., 1995, Daffé et al., 1990). The initial Ara*f* residues are connected *via* α(1 → 5) linkage and are further extended with α(1 → 5) linked Ara*f* (Daffé et al., 1990) residues. The arabinan is a highly branched structure and these branch points are introduced with α(1 → 3) linked Ara*f* residues (Daffé et al., 1990). The chains terminate at the non-reducing ends with a characteristic branched *hexa*-arabinoside unit (Ara*f*_6_), comprising [β-D-Ara*f*-(1 → 2)-α-D-Ara*f*]_2_–3,5-α-D-Ara*f*-(1 → 5)-α-D-Ara*f* (Daffé et al., 1990). The terminal and penultimate Ara*f* residues of the Ara*f*_6_ unit provide the anchoring points for the cell wall bound mycolic acids, of which two thirds are mycolated (Mcneil et al., 1991). Interestingly, some of the 3,5-branch point Ara*f* residues have been found to be modified on the position 2 with either a galactosamine or a succinyl residue (Bhamidi et al., 2008, Draper et al., 1997, Lee et al., 2006, Peng et al., 2012, Škovierová et al., 2010). The purpose of these alterations is not certain, though the additional galactosamine has been found to confer protection from the host’s immune system (Škovierová et al., 2010). Also it is speculated that the galactosamine and succinyl residues could interact to increase the stability of the arabinan domain; the protonated galactosamine has a positive charge and could interact with either the phosphate groups of the membrane lipids or with the negatively charged succinyl residues (Bhamidi et al., 2008).

### 2.5 Arabinogalactan biosynthesis

AG biosynthesis (Fig. 3B) begins in the cytoplasm with the synthesis of the rhamnose-GlcNAc linker unit, α-L-rhamnopyranoside-(1 → 3)-α-D-GlcNAc(1 → P), that connects the AG with the PG. WecA (Rv1302), a GlcNAc-1-P transferase, first produces C_50_-P-P-GlcNAc, using C_50_-P and GlcNAc-1-P (Jin et al., 2010), to which L-rhamnose is added from dTDP-rhamnose, by WbbL (Rv3265c), a rhamnosyltransferase (McNeil et al., 1990, Mills et al., 2004). The decaprenyl-phosphate anchors the resulting rhamnose-GlcNAc linker unit to the inner membrane, with the sugar groups pointing into the cytoplasm. The galactan chain is constructed here using the soluble substrate, UDP-Gal*f*. The dual activity of the galactofuranose transferase, GlfT1 (Rv3782), sequentially polymerises the addition of two priming Gal*f* residues onto the rhamnose of the linker unit (Alderwick et al., 2008, Mikušová et al., 2006): the first Gal*f* is transferred via β(1 → 4) linkage to the D-rhamnose, and the second Gal*f* has β(1 → 5) linkage to the first Gal*f* residue (Belánová et al., 2008). GlfT2 (Rv3808c) then polymerises approximately 28 Gal*f* residues with alternating β(1 → 6) and β(1 → 5) linkages (Kremer et al., 2001a, Rose et al., 2006). At this point, the galactan domain, C_50_-P-P-N-acetylglucosamine-L-rhamnose-galactofuranose_30_, is transported across the inner membrane to the periplasm. There are two candidates for this role, Rv3781 and Rv3783, both of which encode ABC transporters (Dianišková et al., 2011).

The arabinan domain is assembled onto the galactan by membrane-bound arabinofuranosyl transferases (AraT) in the periplasm, using the lipid donor substrate decaprenylphosphoryl-β-D-arabinofuranose (DPA) (Wolucka et al., 1994). The DPA is synthesised in the cytoplasm from ribose-5-phosphate: PrsA (Rv1017c), a ribose-phosphate pyrophosphokinase, first adds a pyrophosphate group from ATP to position C1 of the ribose, to generate 5-phosphoribose-1-diphosphate (pRpp) (Alderwick et al., 2011b). UbiA (Rv3806c) (a decaprenyl-phosphate phosphoribosyltransferase) substitutes the terminal phosphate group from the pyrophosphate for a decaprenyl phosphate (C_50_-P), anchoring it to the inner membrane as decaprenylphosphoryl-β-D-5-phosphoribose (DPPR) (Alderwick et al., 2005, Huang et al., 2008, Huang et al., 2005), and Rv3807c (a putative phospholipid phosphatase) dephosphorylates position C-5 (Jiang et al., 2011). The resulting decaprenylphosphoryl-β-D-ribose (DPR) undergoes an epimerization reaction of the ribose, catalysed by the decaprenylphosphoribose-2′-epimerase, DprE1 (Rv3790) and DprE2 (Rv3791), to produce DPA (Mikušová et al., 2005). The DPA is then flipped across the inner membrane, reorienting the Ara*f* residue into the periplasm. Rv3789 was initially thought to be the DPA transporter (Larrouy-Maumus et al., 2012), though a role in AftA recruitment has also been suggested (Kolly et al., 2015).

In the periplasm, the C_50_-P-P-GlcNAc-L-rhamnose-galactofuranose_30_ (galactan domain) is primed by the AraT, AftA (Rv3792), with three α(1 → 5) linked Ara*f* residues on the C-5 of the β(1 → 6) Gal*f* residues at positions 8, 10, and 12 (Alderwick et al., 2006; 2005). The main body of the arabinan is then constructed by a combination of different AraTs, which introduce straight chains and branch points. Initially, the EmbA (Rv3794)/EmbB (Rv3795) heterodimer was thought to polymerise the longer chains of α(1 → 5) arabinose chains (Alderwick et al., 2005, Khasnobis et al., 2006, Zhang et al., 2020). The role of EmbAB is evidenced by a knock-out of the sole *emb* gene in the related bacteria, *C. glutamicum*, which abolished all AG synthesis, apart from the initial priming Ara*f* (Alderwick et al., 2005). A knock-out of both proteins is lethal in *M. smegmatis*, but a single deletion of either *embA* or *embB*, only demonstrated a role in the construction of the terminal *hexa*-arabinoside unit, which lacked the terminal branch point in the mutants (Escuyer et al., 2001, Khasnobis et al., 2006, Lee et al., 1997). Formation of the terminal branch points, with α(1 → 3) linkage, has since been confirmed with the purified EmbAB enzyme complex (Zhang et al., 2020). It could be that the EmbAB dimer possesses both α(1 → 5) elongation and α(1 → 3) branch point activity, but that the α(1 → 5) activity is redundant in the *embA* or *embB* knock-outs, compensated for by the remaining EmbB or EmbA protein, respectively. Certainly, there is not currently an alternative candidate for the α(1 → 5) elongation. Earlier branch points in the AG are introduced by AftC (Rv2673) and possibly also by AftD (Rv0236c), which incorporate α(1 → 3) linked Ara*f* residues (Birch et al., 2008, Škovierová et al., 2009). Addition of these branches, followed by extensions, leads to a highly branched structure (Alderwick et al., 2005). After the terminal 3,5-branch is formed by EmbAB’s α(1 → 3) transferase activity, AftB (Rv3805c) adds the terminal Ara*f* residues with a β(1 → 2) linkage (Seidel et al., 2007), terminating synthesis with the classic *hexa*-arabinofuranose cap (Seidel et al., 2007). Further modification of the arabinan domain can occur by the addition of a D-galactosamine by Rv3779, or succinyl residue by an unknown transferase, to the C-2 of the terminal 3,5-branched Ara*f* residue (Bhamidi et al., 2008, Draper et al., 1997, Lee et al., 2006, Peng et al., 2012, Škovierová et al., 2010). The completed AG is ligated to PG by the phosphotransferase, Lcp1 (Rv3267) (Harrison et al., 2016).

### 2.6 Drugs targeting arabinogalactan and resistance mechanisms

Ethambutol is a bacteriostatic agent that acts by inhibiting its namesake, the Emb proteins (Belanger et al., 1996), preventing the polymerisation of arabinan, both in AG synthesis by EmbB (Safi et al., 2010, Safi et al., 2008, Starks et al., 2009) and lipoarabinomannan synthesis by EmbC (Goude et al., 2009). Ethambutol inhibition in *M. smegmatis* results in severely truncated arabinogalactan (Deng et al., 1995, Takayama and Kilburn, 1989). In *C. glutamicum*, only the galactan backbone and the priming arabinose residues remain; these are added by AftA, an arabinosyltransferase not affected by ethambutol (Alderwick et al., 2006). Ethambutol treatment also prevented the mycolic acid layer from covalently linking to the cell wall in *M. smegmatis*, due to the absence of attachment sites on the AG (Takayama and Kilburn, 1989). Recent structural studies have described ethambutol bound to the active sites of EmbB and EmbC, inhibiting activity by competing with the arabinose of both the donor and acceptor substrates (PDB IDs: 7BVC, 7BWR, 7BVG, 7BVF, 7BVE and 7BVH) (Zhang et al., 2020). Interestingly, all three Emb proteins co-purified with the FAS-II acyl carrier protein, AcpM, bound to their cytoplasmic exposed surface, though more work is required to determine the function of this protein in AG/LAM assembly (Zhang et al., 2020). Resistance to ethambutol predominantly occurs with mutations in EmbB (93.7%), most of which are substitutions of methionine-306 to a branched chain amino acid (isoleucine, leucine or valine) (Zhao et al., 2015). 72.7% of all MDR strains identified have mutations in EmbB, which reflects the universal application of ethambutol as a front-line drug (Zhao et al., 2015).

The nitrobenzamine drug, BTZ, has uncovered DprE1 as another useful target in the AG pathway; this drug is active in low doses against both MDR and XDR strains (Makarov et al., 2009, Pasca et al., 2010). DprE1 acts as an epimerase along with DprE2, converting DPR to DPA, the substrate used by all arabinosyltransferases in *Mtb* (Wolucka et al., 1994). Inhibition occurs when the nitro group of BTZ is converted by DprE1 to a nitroso group, which then forms a covalent bond with the active site cysteine-387 (Batt et al., 2012, Makarov et al., 2009, Neres et al., 2012, Trefzer et al., 2010). Though resistance can develop through the substitution of this cysteine for a serine (Makarov et al., 2009), there are several inhibitors in the pipe-line, including TCA1 (Wang et al., 2013), GSK710 (Batt et al., 2016) and Azaindole (Chatterji et al., 2014), that do not rely on cysteine-387 for inhibition.

The caprazamycin derivative, CPZEN-45, inhibits WecA (Ishizaki et al., 2013), an essential protein involved in the synthesis of the unique rhamnose-GlcNAc linker between AG and PG (Huszár et al., 2017, Jin et al., 2010). While CPZEN-45 has good pharmacological properties and is active against MDR strains, it has poor bioavailability and so is currently being trialled for inhalation therapy in combination with the unrelated second-line drug, capreomycin (Pitner et al., 2019). The substrate UDP-Gal*f* is not utilised in humans (Peltier et al., 2008, Tefsen et al., 2012) and so substrate analogues could be exploited to target the galactofuranosyltransferases, GlfT1 and GlfT2, although none to date have sufficient activity against the enzyme or whole cell (Konyariková et al., 2020).

### 2.7 Structures of the mycolic acids

Mycolic acids (MA), an abundant lipid of *Mtb*, are covalently attached to two-thirds of the non-reducing ends of the AG, where they extend out, perpendicular to the inner membrane, forming the inner leaflet of the MOM (Mcneil et al., 1991, Minnikin, 1982). Since they are long chains of 70–90 carbons (α-alkyl-β-hydroxy fatty acids), the MAs fold into energetically favourable conformations, which is dependent on their structural features. This enables tight packing within the layer, forming an impermeable hydrophobic barrier, which is essential for viability (Bhatt et al., 2005, Brown et al., 2007) and pathogenesis (Dubnau et al., 2000, Glickman et al., 2000, Peyron et al., 2008, Rao et al., 2006, Yuan et al., 1998), while also preventing antibiotic entry into the cell (Jarlier and Nikaido, 1994, Liu and Nikaido, 1999). While some MAs are linked to the cell wall, others are present as extractable lipids in the outer leaflet of the MOM. The majority of the extractable MAs are linked to trehalose as trehalose monomycolates (TMM) and trehalose dimycolates (TDM), though free mycolates are also present, particularly in latent phase cells (Bacon et al., 2014). TMM and TDM are thought to be intermediates in the formation of the MA layer (Kremer and Besra, 2005), though TDM, the ‘cord factor’, also has roles in pathogenicity. TDM interacts with the macrophage receptor Mincle, a C-type lectin receptor, preventing acidification of the phagosome and enabling granuloma formation (Hunter et al., 2006, Indrigo et al., 2003, Ishikawa et al., 2009, Patin et al., 2017). The free mycolates have also been found to promote granuloma formation, which correlates with their abundance in latent cells (Bacon et al., 2014).

**Fig. 4:**
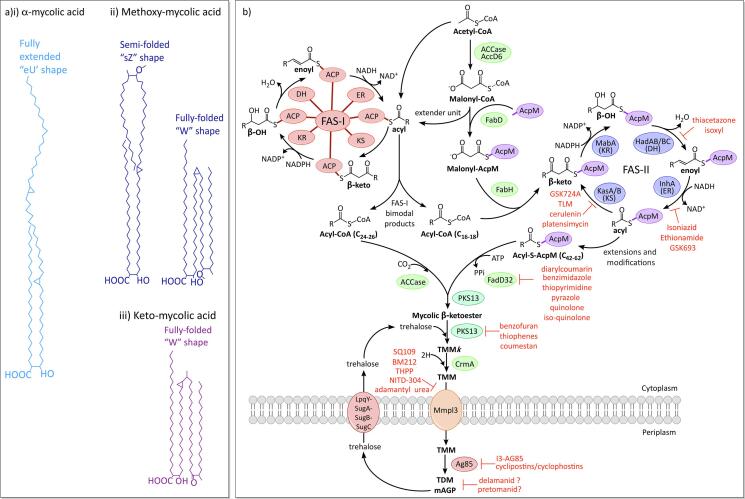
Chemical features of mycolic acids and biosynthesis A) Structures and common conformations of the three classes of MAs: i) α-, ii) Methoxy- and iii) Keto- (Minnikin et al., 2015). B) Mycolic acid biosynthesis.

MAs are formed of two parts that can be cleaved at high temperatures: the meromycolate moiety is a long chain meroaldehyde of up to 62 carbons, usually with two functional groups, including cyclopropane rings, methyl groups and oxygen functions (Asselineau and Lederer, 1950; Barry et al., 1998). The α-branch is a shorter, saturated chain of 24–26 carbons, without functional groups (Asselineau and Lederer, 1950). The three classes of MAs in *Mtb* (Fig. 4A) consist of α-mycolates, methoxymycolates and ketomycolates. The α-MAs are the most abundant and contain only *cis*-cyclopropane rings, while the oxygenated methoxy-MAs and keto-MAs have either a *cis*-cyclopropane ring, or a *trans-*cyclopropane ring with an adjacent methyl branch (Brennan and Nikaido, 1995, Minnikin, 1982). Methoxy-MAs are more abundant with *cis*-cyclopropane rings, while keto-MAs are more common with *trans*-cyclopropane rings (Watanabe et al., 2002). The cyclopropane rings introduce kinks into the long chains (Brennan and Nikaido, 1995), which along with hydrophilic interactions from the oxygen functions (Villeneuve et al., 2005), enables the MAs to fold into different conformational shapes. The keto-MAs typically adopt a ‘W’ shape, packing together four chains in parallel (Villeneuve et al., 2007; 2005), while the α- and methoxy-MAs are more flexible and can take on more open structures (Groenewald et al., 2014). Interestingly, the ‘W’ conformations of the keto-MAs are reliant on the *trans*-configuration of the cyclopropane ring, a feature that could support a more tightly packed MA layer in strains with a higher proportion of *trans*-rings (Villeneuve et al., 2013) and could explain the requirement of keto-MAs for virulence (Sambandan et al., 2013, Yuan et al., 1998).

### 2.8 Mycolic acid biosynthesis

Fatty acid synthase-I (FAS-I) (*fas*; Rv2524c) is the only system in *Mtb* capable of synthesising fatty acids *de novo*. Uniquely to mycobacteria, the product distribution is bimodal, and the synthesised fatty acids can be one of two different chain lengths: either longer, C_24_-C_26_ chains, or shorter chains of C_16_-C_18_ (Bloch and Vance, 1977, Peterson and Bloch, 1977). In MA synthesis, the C_24_-C_26_ chains produce the α-branch, while the shorter chains are elongated by FAS-II to form the meromycolate moiety. FAS-I is a ‘eukaryotic-like’ multi-domain enzyme, a single protein with all the catalytic domains for fatty acid synthesis: acyl transferase (AT), enoyl reductase (ER), dehydratase (DH), malonyl/palmitoyl transferase (MPT), acyl carrier protein (ACP), β-keto reductase (KR) and β-ketoacyl synthase (KS) (Fernandes and Kolattukudy, 1996). Synthesis commences with the transfer of an acetate from Coenzyme A (CoA) to the ACP domain (Fig. 4B). During each cycle the chain is extended by two carbons (acetate) from the extender unit, malonyl-CoA, which is produced through the carboxylation of acetyl-CoA by the acyl-CoA carboxylase complex (ACCase) (Cronan and Waldrop, 2002). The mechanism employed to produce fatty acid products with two quite different chain lengths, is complex and could involve interactions between several components of both FAS systems. Also implicated in this are endogenous polysaccharides, containing either 3-O-methyl-D-mannose or 6-O-methyl-D-glucose, which have been shown to favour the shorter chain lengths by complexing with these products and facilitating their release from the FAS-I complex (Banis et al., 1977, Papaioannou et al., 2007, Wood et al., 1977).

FAS-II is a ‘prokaryote-like’ fatty acid synthase, a complex of discrete enzymes that correspond to the catalytic domains of FAS-I. The extender unit, malonyl is presented on an acyl carrier protein (AcpM; Rv2244), which is transferred from malonyl-CoA by the transacylase, FabD (Rv2243) (Kremer et al., 2001b). FabH (Rv0533c), a β-ketoacyl ACP synthase, shuttles the shorter C_16_-C_18_ acyl-CoA chains from FAS-I to FAS-II, through a Claisen-like condensation with malonyl-AcpM (Choi et al., 2000). The resulting β-ketoacyl-AcpM is presented to the FAS-II enzymes, where the keto group is reduced by the keto-reductase, MabA (Rv1483), to form β-hydroxylacyl-AcpM (Marrakchi et al., 2002). The dehydratase heterodimers, HadAB (Rv0635-Rv0636) and HadBC (Rv0636-Rv0637) convert this to enoyl-AcpM (Brown et al., 2007, Sacco et al., 2007), which is then reduced to acyl-AcpM by the enoyl-CoA reductase, InhA (Rv1484) (Banerjee et al., 1994). Further rounds are then initiated by the β-ketoacyl synthases, KasA/B (Rv2245 and Rv2246), extending the chain by an acetate each cycle (Kremer et al., 2002a, Schaeffer et al., 2001). FAS-II extends the acyl chain to C_18_-C_32_, after which modifications, such as further extensions to C_42_-C_62_, *cis*-/*trans*- cyclopropanations, methylations and methoxy-/keto-group additions, produce the mature meromycolate chain (Barkan et al., 2010, Glickman, 2003, Glickman et al., 2000; Barry et al., 1998).

Pks13 (Rv3800c) is an iterative Type I polyketide synthase (PKS) that joins together the two branches of the MAs through a Claisen-like condensation (Portevin et al., 2004). This involves the action of FadD32 (Rv3801c), a fatty-acyl-AMP ligase, which activates the meromycolyl-AcpM to meromycolyl-AMP and loads the acyl chain onto the N-terminal ACP of Pks13 (Léger et al., 2009, Trivedi et al., 2004). The α-branch is carboxylated by the ACCase complex, a step that is necessary for the Claisen-type reaction (Gande et al., 2007), and loaded onto the C-terminal ACP domain of Pks13 (Gavalda et al., 2014). The product of the condensation reaction is then transferred onto a trehalose by Pks13, producing α-alkyl β-ketoacyl trehalose monomycolate (TMM*k*) (Gavalda et al., 2014) and CmrA (Rv2509) reduces the keto group to produce the mature trehalose monomycolate (TMM) (Bhatt et al., 2008, Lea-Smith et al., 2007). The TMM is transported across the inner membrane by MmpL3 (Rv0206c) (Grzegorzewicz et al., 2012, Tahlan et al., 2012). In the periplasm, a complex of secreted proteins with mycolyltransferase activity, known as the Antigen 85 complex (Ag85A, Ag85B, Ag85C; Rv3804c, Rv1886c, Rv0129c), attach the TMM either directly to the AG to form mAG (mycolylarabinogalactan), or to an acceptor TMM, producing TDM (Belisle et al., 2009, Jackson et al., 1999). These processes release trehalose, a significant sugar for *Mtb*, which is recycled back into the cell by LpqY-SugA-SugB-SugC (Rv1235-Rv1236-Rv1237-Rv1238), an essential ABC sugar transporter (Kalscheuer et al., 2010).

### 2.9 Drugs targeting mycolic acid biosynthesis and resistance mechanisms

MAs are essential for viability and as such the proteins involved in MA biosynthesis and transport represent excellent drug targets (Bhatt et al., 2005, Brown et al., 2007). The front-line drug, isoniazid (INH), and the structurally related second-line drug, ethionamide (ETH), inhibit the FAS-II enoyl-CoA reductase, InhA (Banerjee et al., 1994). INH is a pro-drug that must first be activated by the peroxidase activity of KatG (Rv1908c) (Zhang et al., 1992), forming an INH-NAD adduct that competitively inhibits InhA and stalls MA synthesis (Rawat et al., 2003). Isoniazid has been used to treat tuberculosis since 1952 (Murray et al., 2015) and as such resistance has developed with up to 82% of all MDR isolates having mutations in the *katG* gene (Torres et al., 2015), which prevents activation of the pro-drug; other resistance mechanisms include mutations in the NADH binding pocket of InhA (Banerjee et al., 1994, Dessen et al., 1995, Rozwarski et al., 1998), which have the additional downside of ETH cross-resistance. ETH is also a pro-drug, though is activated by an alternative mechanism, EthA (Rv3854c), a monooxygenase that oxidises ETH to the active species (Baulard et al., 2000, DeBarber et al., 2000, Vannelli et al., 2002); mutations in EthA have been identified in up to 76% of ETH-resistant isolates (Morlock et al., 2003). The search for further InhA inhibitors, through compound library screening for activity against InhA, has revealed a new set of thiadiazole inhibitors, the most promising of which is GSK693; this does not require activation by KatG and so bypasses this route of resistance, and is active against MDR and XDR isolates (Martínez-Hoyos et al., 2016). The *in vitro* resistance mutation rate of GSK693 is also much lower than that of INH and it is hoped that this will represent a lower frequency of spontaneous resistant mutants in clinical isolates; any resistance mutations map to the active site of the *inhA* gene (glycine-96 or methionine-103) (Martínez-Hoyos et al., 2016).

The β-ketoacyl synthases, KasA/B, are another useful drug target in the mycolic acid pathway, with inhibitors including thiolactomycin (Kremer et al., 2000), although activity against the whole cell is poor (Miyakawa et al., 1982); cerulenin (Schaeffer et al., 2001); and platensimycin (Brown et al., 2009). Additionally, an indazole sulfonamide, GSK724, which targets just KasA, has been found through whole cell screening and generation of spontaneous resistant mutants in *M. bovis* BCG (Abrahams et al., 2016). Interestingly, the co-crystal structure of KasA with GSK724 has demonstrated that the inhibitor binds to the acyl channel, which contrasts to the binding observed for other inhibitors of KasA (PDB ID: 5LD8) (Abrahams et al., 2016). Isoxyl and thiacetazone, thiocarbamide-containing drugs previously used in the treatment of tuberculosis, are pro-drugs that are activated by EthA, and ultimately inhibit the FAS-II dehydratase, HadAB (Grzegorzewicz et al., 2015). Although discontinued due to their toxic side effects, they nonetheless have validated HadAB to be another useful drug target.

FadD32, which is involved in the condensation of the two MA branches, is essential in *Mtb* (Portevin et al., 2005). Diarylcoumarins were initially identified as effective inhibitors that are also active in *Mtb* whole cell and animal models (Stanley et al., 2013), and more chemically stable derivatives have been synthesised with the addition of a quinoline ring (Fang et al., 2018). High-throughput screening (HTS) using an assay with purified FadD32 located a further five classes of inhibitor: thiopyrimidine, benzimidazole, pyrrozole, quinolone and *iso*-quinolone (Galandrin et al., 2013).

PKS13, the polyketide synthase that condenses the two branches of the MA, is also an essential target (Portevin et al., 2004, Wilson et al., 2013), with inhibitors including benzofuran, thiophenes and coumestan. The benzofuran, TAM16, which is active against MDR and XDR strains, inhibits PKS13 by binding to the active site of the thioesterase (TE) domain (Aggarwal et al., 2017). Coumestan analogues are derived from modifications of benzofuran and similarly inhibit the TE domain (Zhang et al., 2018). Thiophenes have a different mechanism to benzofurans, binding to the N-terminal ACP domain and preventing interactions with FadD32 (Wilson et al., 2013).

The generation of spontaneous resistant mutants to many of the recently available drugs found to target *Mtb*, has identified MmpL3 as a potentially significant new drug target (Grzegorzewicz et al., 2012, Tahlan et al., 2012). The MmpL proteins are RND (resistance, nodulation and cell division) superfamily membrane proteins (Saier et al., 1994), involved in lipid transport across the inner membrane. MmpL3 transports the MA precursor, TMM, and is the only essential MmpL protein in *Mtb* (Domenech et al., 2005). The structural diversity of these new drugs, along with their broad-spectrum of targets in other bacterial and fungal species, has caused some ambiguity as to their true target in *Mtb*, though many have been demonstrated to bind directly to MmpL3 in the crystal structure, including SQ109 (1,2-ethylenediamine), AU1235 (adamantyl urea) and ICA (indolcarboxamide) (PDB IDs: 6AJH, 6AJJ, 6AJI, 6AJF and 6AJG) (Zhang et al., 2019). In this study, the compounds that were either co-crystallised or modelled to the MmpL3 structure, bound to the same part of the central channel involved in proton relay, disrupting critical interactions between aspartic acid and tyrosine residues (Zhang et al., 2019). The most promising MmpL3 inhibitor is SQ109, which is currently in phase II clinical trials in the USA (World Health Organisation, 2019).

The secreted complex, AG-85, responsible for mycolic acid attachment and TDM synthesis, consists of three related proteins, though there is some redundancy (Belisle et al., 1997, Jackson et al., 1999): the loss of Ag85B is tolerated, though Ag85A is required for growth in macrophages (Armitige et al., 2000) and a deficiency in Ag85C reduces mycolic acid transfer to the mAGP complex by 40% (Jackson et al., 1999). I3-AG85 (2-amino-6-propyl-4,5,6,7-tetrahydro-1-benzothiophene-3-carbonitrile) is a derivative of a compound from a library found to inhibit Ag85C, which is active against MDR and XDR strains, though the MIC is poor (Warrier et al., 2012). Potent analogues of I3-AG85 derivatives have been produced using fragment-based drug discovery (Scheich et al., 2010). Cyclipostins/cyclophostins are a new class of monocyclic-enolphosphonate that bind covalently to the catalytic serine residue of Ag85C and are potent against *Mtb* (Viljoen et al., 2018). Ebselen similarly binds covalently to an active site cysteine in Ag85C and is active against MDR strains (Favrot et al., 2014).

Mycolic acid attachment to AG is also inhibited by the second-line drugs, pretomanid and delamanid, though the exact targets are still not known (Matsumoto et al., 2006, Stover et al., 2000). Resistant mutants have thus far only been generated in *ddn*, encoding a deazaflavin dependent reductase, and enzymes involved in the synthesis and reduction of the F_420_ cofactor (Choi et al., 2001, Manjunatha et al., 2006), which is thought to be a drug activation system rather than the target (Haver et al., 2015, Hoffmann et al., 2016, Manjunatha et al., 2006).

### 2.10 Phosphatidyl-myo-inositol mannosides (PIMs), lipomannan (LM) and lipoarabinomannan (LAM)

**Fig. 5:**
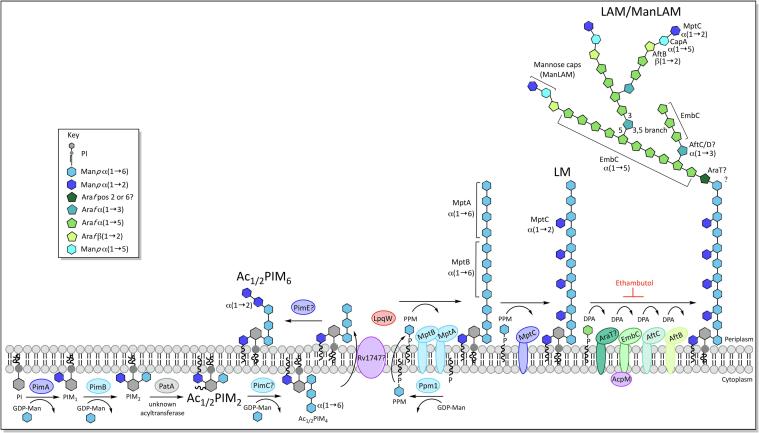
Biosynthesis of phosphatidyl-inositol-mannosides (PIMs), lipomannan (LM) and lipoarabinomannan (LAM).

Another distinctive feature of the mycobacterial cell wall is the presence of three species of glycolipids: phosphatidyl-*myo*-inositol mannosides (PIMs), lipomannan (LM) and lipoarabinomannan (LAM), which have been found in abundance, non-covalently bound to the inner membrane, and possibly also the outer membrane (Ortalo-Magné et al., 1996, Pitarque et al., 2008). The core structure consists of an acylated *sn*-glycero-3-phospho-(1-D-*myo*-inositol) (PI) unit, glycosylated with up to six α-D-mannopyranosyl (Man*p*) residues in PIMs and a longer Man*p* core in LM/LAM (Kaur et al., 2007, Mishra et al., 2007), with an additional highly branched arabinan domain in LAM (Fig. 5). In eukaryotes, various derivatives of the PI unit exist, which are often involved in cell signalling (Falasca and Maffucci, 2006, Kutateladze, 2006, Lindmo and Stenmark, 2006). The mycobacterial PI unit differs in that it is glycosylated with Man*p* residues at the O-2 and O-6 positions of the inositol ring, forming a mannosyl phosphate inositol (MPI) anchor. PIMs exist in varying degrees of mannosylation, carrying 1 to 6 Man*p* residues, though tri- and tetra-acylated phospho-*myo-*inositol dimannosides (Ac_1_PIM_2_ and Ac_2_PIM_2_) and hexamannosides (Ac_1_PIM_6_ and Ac_2_PIM_6_) are the most prevalent forms of PIMs in *M. bovis* BCG, *M. tuberculosis* H37Rv and *M. smegmatis* (Khoo et al., 1995a). Both forms have been shown to be an important structural feature of the inner membrane, increasing stability and decreasing permeability to drugs (Bansal-Mutalik and Nikaido, 2014). In contrast to the most abundant PIMs, LM and LAM are highly mannosylated, commonly carrying a chain of 21–34 α(1 → 6) linked Man*p* residues, interspersed with 5–10 single branched α(1 → 2) linked Man*p* units (Chatterjee et al., 1992a). LAM has an additional highly branched arabinan layer of 50–80 arabinofuranose (Ara*f*) residues, similar to the domain on AG (Khoo et al., 1996).

LAM and LM both play an important role in the modulation of the host’s immune defences (Schlesinger et al., 1994, Shukla et al., 2018). A variety of LAM caps exist, each affecting the immune response in different ways. Mannose capped LAM (ManLAM) is a major *Mtb* virulence factor, which is thought to contribute to the inhibition of the host’s immune response, and also to act as the ligand for phagocytosis of *Mtb* (Schlesinger et al., 1994). Conversely, *Mtb* has been shown to stimulate an immune response through toll-like receptor 2 (TLR2), signalling *via* phosphoinositol-capped LAM (PILAM) and LM, which both act as TLR2 agonists (Shukla et al., 2018). For these interactions to occur, LM and LAM would need to be surface exposed, and indeed it has been shown these lipoglycans insert into the outer membrane lipids, in addition to those of the inner membrane (Ortalo-Magne et al., 1996, Pitarque et al., 2008, Sani et al., 2010). However, the issue of LM/LAM location is still disputed, with other studies only demonstrating surface exposure in the presence of the cell wall inhibitors, isoniazid and ethambutol (Alsteens et al., 2008). Also, an outer membrane location would require an as yet undiscovered transport system, though a system similar to that of *E. coli* LPS transport has been suggested (Pitarque et al., 2008). Indeed, most immunomodulatory studies have relied upon purified components, which does not account for surface exposure (Fratti et al., 2003, Knutson et al., 1998, Nigou et al., 2001, Vergne et al., 2003), though an interaction between ManLAM and the host’s immune system has been confirmed using live *Mtb* (Decout et al., 2018).

### 2.11 Biosynthesis of phosphatidyl-myo-inositol mannosides, lipomannan and lipoarabinomannan

The synthesis of these lipoglycans progresses from PIM → LM → LAM (Besra et al., 1997) (Fig. 5) and begins in the cytoplasm with the production of *sn*-glycero-3-phospho-(1-D-*myo*-inositol), a phosphatidylinositol (PI) anchored to the inner membrane by two fatty acid chains linked to the glycerol moiety. This occurs by way of a two-part process: PgsA1 (Rv2612c), a phosphatidylinositol phosphate synthase, initiates the biosynthesis of PI by catalysing the conjugation of D-*myo*-inositol-3-phosphate with cytidine diphosphate diacylglycerol (Jackson et al., 2000, Morii et al., 2010). The phosphatidylinositol phosphate formed in this reaction is then broken down into phosphatidylinositol and phosphate by an unknown phosphatase (Grāve et al., 2019, Jackson et al., 2000, Morii et al., 2010).

A group of GDP-mannopyranose (Man*p*) dependent cytoplasmic α-mannopyranosyltransferases (Man*p*T), from the GT-A/B (glycosyltransferase A/B) superfamily (Liu and Mushegian, 2003, Morita et al., 2005), are involved in the early stages of PIM biosynthesis, building upon the *myo*-inositol ring of the PI, which is anchored on the cytoplasmic face of the inner membrane: PimA (Rv2610c) transfers the first Man*p* to the O-2 position with α(1 → 2) linkage, forming PIM_1_ (Boldrin et al., 2014, Guerin et al., 2007, Korduláková et al., 2002a) and PimB (Rv2188c) transfers a second Man*p* residue with α(1 → 6) linkage to position O-6 to form PIM_2_ (Guerin et al., 2009). An acyltransferase, Rv2611c, acylates position C-6 of the Man*p* residue at position 2 (Korduláková et al., 2003). It is not entirely clear whether this acylation occurs before or after the mannosylation of PIM_1_, though studies have shown that Ac_1_PIM_1_ is preferentially formed over Ac_1_PIM_2_, suggesting that PIM_1_ is the likely acceptor (Korduláková et al., 2003). An unknown transferase acylates the C-3 position of the *myo-*inositol ring, to form the tetra-acylated mannosylated PI anchor (MPI anchor), though the tri-acylated form is more abundant (Khoo et al., 1995a). The third and fourth Man*p* residues are added with α(1 → 6) linkage, to the Man*p* at position 6 of the inositol ring, forming Ac_1/2_PIM_3_ and Ac_1/2_PIM_4_ respectively. The Man*p*T(s) responsible for this has yet to be determined; PimC has been proposed to transfer the first or both of these residues, though there appears to be multiple pathways, and while PimC is present in *M. tuberculosis* CDC1551, other mycobacterial species such as *Mtb* H37Rv and *M. smegmatis* do not carry a homologous gene (Kremer et al., 2002b).

At some point in the synthesis between Ac_1/2_PIM_2_-Ac_1/2_PIM_4_, the mannosylated inositol moiety is transferred across the membrane and into the periplasm, possibly by the ABC transporter, Rv1747, though there is some redundancy in the pathway (Glass et al., 2017). Here, the synthesis of the higher PIMs, LM and LAM is carried out by membrane-bound GT-C superfamily Man*p*Ts (Liu and Mushegian, 2003, Morita et al., 2005) using polyprenyl-phosphate-based mannose donors, which are synthesised by Ppm1 (Rv2051c) (Berg et al., 2007, Gurcha et al., 2002). Ac_1/2_PIM_4_ forms the branch point between the Ac_1/2_PIM_6_ pathway and LM/LAM synthesis. This is mediated by the lipoprotein, LpqW (Rv1166) (Kovacevic et al., 2006), knock-outs of which have shown that LpqW favours the synthesis of LM/LAM by enhancing the activity of the subsequent mannosyltransferase in their synthesis, MptB (Crellin et al., 2008, Rainczuk et al., 2012). PimE (Rv1159) facilitates the PIM_6_ pathway, adding the α(1 → 2) linked fifth and possibly sixth mannose residues to the chain of three Man*p* residues at position 6 of the inositol ring, forming Ac_1/2_PIM_5_, followed by Ac_1/2_PIM_6_ (Morita et al., 2006).

The linear mannose core of LM is also elongated from the chain of Man*p* residues present at position 6 of the inositol ring. MptA (Rv2174) and MptB (Rv1459c) add further α(1 → 6) linked Man*p* residues here and knockouts have demonstrated that MptB adds the initial part of the chain, followed by MptA (Mishra et al., 2008; 2007). The mature LM possesses additional mannose residues arranged as α(1 → 2) linked monomannose side chains, which are transferred by MptC (Rv2181) (Kaur et al., 2008, Mishra et al., 2011).

LAM synthesis builds upon a mature LM core, adding a highly branched arabinan domain, which is synthesised by membrane-bound arabinofuranosyltransferases (AraTs), using DPA (decaprenylphosphoryl-β-D-arabinofuranose) as the arabinose donor (Wolucka et al., 1994). The mannose core is first primed with Ara*f* (arabinofuranose), by an unknown AraT, in what is thought to be a similar process to the synthesis of the arabinogalactan domain. The position of this priming is not clear and both the O-2 (Chatterjee et al., 1993) and O-6 (Angala et al., 2016) positions on the mannose have been implicated, the latter of which would prevent attachment of the arabinan domain to all but the last mannose residue in the core. EmbC (Rv3793) then elongates the primed core, adding 12–16 α(1 → 5) linked Ara*f* residues (Alderwick et al., 2011a, Shi et al., 2006). Branching is introduced into the linear arabinan chain by AftC (Rv2673), which adds α(1 → 3) linked Ara*f* residues (Birch et al., 2008). It has been postulated that AftD (Rv0236c) initiates further α(1 → 3) branching, although this is unconfirmed (Škovierová et al., 2009). AftB (Rv3805c) terminates the arabinan with β(1 → 2) linked Ara*f* (Jankute et al., 2017). The structure of the arabinan domain, by this point is highly branched, though the non-reducing ends are less branched than those of AG, with a linear tetra-arabinoside motif more common than the branched *hexa*-arabinoside of AG (Chatterjee et al., 1993). CapA (Rv1635c), a PPM dependent α(1 → 5) Man*p*T, primes the arabinan termini for capping by adding a single Man*p* residue (Dinadayala et al., 2006). MptC is responsible for further mannosylation of the cap through the additions of one to three α(1 → 2) Man*p* residues (Kaur et al., 2008). In *M. tuberculosis*, a substitution occurs in approximately 15–20% of mannose caps replacing mannose with a single α-(1 → 4) linked methylthio-D-xylose residue (Angala et al., 2017, Ludwiczak et al., 2002, Turnbull et al., 2004). The decoration of LAM varies between mycobacterial species: while *Mtb* and other slow-growing pathogenic strains have up to three mannose residues (Chatterjee et al., 1992b), phospho-inositol (PI) capped LAM (PILAM) is more common in non-pathogenic species, such as *M. smegmatis* (Khoo et al., 1995b); some rapid-growing species such as *M. chelonae* lack mannose and PI caps entirely (Guerardel et al., 2002).

### 2.12 Drugs targeting the biosynthesis phosphatidyl-myo-inositol mannosides, lipomannan and lipoarabinomannan and the mechanisms of resistance

Although strains expressing truncated forms of LAM are viable (Goude et al., 2009), no strains completely lacking LAM have been grown, indicating a role in viability (Korkegian et al., 2014). This is confirmed by the essentiality of the major arabinosyltransferase involved in LAM synthesis, EmbC, in *Mtb* (Goude et al., 2008). Analogously to EmbB, EmbC is inhibited by the front-line drug ethambutol (Goude et al., 2009), and structural studies have demonstrated ethambutol binding to the active site of EmbC (PDB ID: 7BVE) (Zhang et al., 2020). Although, EmbB is considered the primary target of ethambutol, treatment also results in truncated LAM, and overexpression of EmbC can confer resistance to ethambutol, indicating that inhibition of LAM biosynthesis is a part of ethambutol activity (Goude et al., 2009). Interestingly, the structure of EmbC has recently demonstrated an association with the FAS-II protein AcpM, as also seen with EmbAB. While the function of AcpM with these arabinosyltransferases is not clear, mutating the amino acid side chains of EmbC involved in these contacts, reduced the LAM content, suggesting a role in synthesis and activity (Zhang et al., 2020). DprE1 is critical to the synthesis of the arabinosyltransferase donor, DPA, and so the inhibitors of this enzyme, which were discussed earlier in relation to AG, would also prevent synthesis of the arabinan domain of LAM (Makarov et al., 2009).

Many of the drugs targeting the LM/LAM pathway have so far been substrate analogues, with targets including PimA, PimB and Ppm1 (Brown et al., 2001, Dinev et al., 2007, Subramaniam et al., 2005), known to be essential enzymes in either *M. smegmatis* or *Mtb* (Korduláková et al., 2002b, Rana et al., 2012, Torrelles et al., 2009). A galactose phosphonate analogue of PI was found to inhibit PimA in a cell-free, but not a whole-cell assay (Dinev et al., 2007). More recently, however, there has not been much progress in the way of new inhibitors of this pathway, which could reflect a reduced role of LM/LAM in *Mtb* viability, as compared to the mAGP complex, or a deficiency of research into inhibitors of pathogenicity.

## 3 Conclusions

The cell wall of mycobacteria is essential for viability and virulence, and the complex pathways responsible for its synthesis contain a plethora of essential enzymes, which could be targets for new antibiotics. Currently, although there is a great deal of research into drugs and targets for *Mtb*, most new drugs are initially found through whole cell assays, and the targets later located through spontaneous mutant generation. This process is lengthy and prone to error, with mutations often generated in drug activation pathways (Haver et al., 2015, Hoffmann et al., 2016, Juréen et al., 2008, Manjunatha et al., 2006, Morlock et al., 2003, Stoffels et al., 2012, Torres et al., 2015), and efflux pumps (such as MmpL5 and MmpL7) (Halloum et al., 2017, Hartkoorn et al., 2014, Milano et al., 2009). Indeed, the targets of many drugs, such as pretomanid, delamanid and pyrazinamide, are yet to be found.

Future research in drug discovery will likely take a more targeted approach as our understanding of this complex pathogen improves: new targets will be chosen due to their uniqueness and essentiality; target-based enzyme assays will be developed on a HTS basis to rapidly screen large compound libraries; and structural studies and modelling will be used to improve the properties and binding of any identified inhibitors. Examples of this shift in research methods have been touched upon here. One such instance is the GlaxoSmith Kline (GSK) library screen against the validated old target, InhA, revealing a new set of inhibitors, the thiadiazoles (Martínez-Hoyos et al., 2016). Additionally, HTS assays have also been developed for new targets, such as WbbL (Grzegorzewicz et al., 2008), WecA (Mitachi et al., 2016), FadD32 (Galandrin et al., 2013) and Ag85 (Boucau et al., 2009), so that drug libraries can be screened specifically for inhibition of these targets. One downside to these *in vitro* screening methods, that whole cell activity is not demonstrated, is particularly significant for *Mtb*, which has such an impenetrable hydrophobic barrier to antibiotics in the form of its unique cell wall; though this could be easily remedied by first screening libraries for activity against the whole cell. Another useful targeted approach to drug development is whole-cell target overexpression, which has been used to find a novel inhibitor of DprE1, GSK710 (Batt et al., 2016). This technique overexpresses the target protein in *Mtb* or related bacteria and identifies inhibitors as having an associated increase in the concentration of compound required to inhibit the cell; it has the added benefit of demonstrating activity not only against the target protein, but the whole cell too.

Structural research, even of membrane proteins, has exploded in recent years; this has been aided by advances in technology, particularly cryo-EM, which now has resolution that rivals X-ray crystallography. Cryo-EM has been used to resolve the structures of the targets of the front-line drug ethambutol, the Emb proteins, demonstrating both the inhibitor binding mechanism and Emb co-localisation with the FAS-II acyl carrier protein, AcpM (Zhang et al., 2020). Crystal studies have also revealed the structure of the exiting new target MmpL3, the membrane protein involved in mycolic acid transport and the target of many new antibiotics under development (Zhang et al., 2019). The MmpL proteins are involved in lipid transport across the inner membrane, an important process in *Mtb*. While MmpL3 is the only essential MmpL protein, others are important for virulence (Domenech et al., 2005) and should not be discounted as useful targets in the future.

Despite the increased efforts into drug discovery, only two new antibiotics have been approved in the last 40 years, bedaquiline and delamanid (Andries et al., 2005, Thakare et al., 2015). Hopefully, as our insight into the mechanisms of this powerful pathogen improves, a more targeted approach should be more productive in the discovery and approval of new antibiotics.

## 4 Author statements

### 4.1 Ethics statement

No ethical issues to report.

## CRediT authorship contribution statement

**Sarah M. Batt:** Conceptualization, Writing - original draft, Writing - review & editing, Visualization. **Christopher Burke:** Conceptualization, Writing - original draft, Writing - review & editing, Visualization. **Alice Moorey:** Conceptualization, Writing - original draft, Writing - review & editing, Visualization. **Gurdyal S. Besra:** Conceptualization, Writing - original draft, Writing - review & editing.

## Declaration of Competing Interest

The authors declare that they have no known competing financial interests or personal relationships that could have appeared to influence the work reported in this paper.
